# Proteins pattern alteration in AZT-treated K562 cells detected by two-dimensional gel electrophoresis and peptide mass fingerprinting

**DOI:** 10.1186/1477-5956-4-4

**Published:** 2006-03-29

**Authors:** Gabriele D'Andrea, Anna R Lizzi, Sara Venditti, Laura Di Francesco, Alessandra Giorgi, Giuseppina Mignogna, Arduino Oratore, Argante Bozzi

**Affiliations:** 1 Department of Biochemical Sciences and Technologies, University of L'Aquila, Via Vetoio, 67100 L'Aquila, Italy; 2 Department of Biochemical Sciences, University La Sapienza, P.le Aldo Moro 5, 00185 Rome, Italy

## Abstract

In this study we report the effect of AZT on the whole protein expression profile both in the control and the AZT-treated K562 cells, evidenced by two-dimensional gel electrophoresis and peptide mass fingerprinting analysis. Two-dimensional gels computer digital image analysis showed two spots that appeared up-regulated in AZT-treated cells and one spot present only in the drug exposed samples. Upon extraction and analysis by peptide mass fingerprinting, the first two spots were identified as PDI-A3 and stathmin, while the third one was proved to be NDPK-A. Conversely, two protein spots were present only in the untreated K562 cells, and were identified as SOD1 and HSP-60, respectively.

## Background

AZT (3'-azido-3'-deoxythymidine or zidovudine), the first anti-retroviral drug approved for AIDS therapy, is a synthetic nucleoside analogue that inhibits HIV reverse transcriptase activity in vitro [1]. It is often included as one of the best anti-HIV 'drugs of choice' in highly active anti-retroviral therapy (HAART) together with other non-nucleoside analogues and protease inhibitors [2-5]. Besides its inhibitory effect on reverse transcriptase, AZT is known to play a key role in many other cellular processes (e.g. protein and lipid glycosylation [6-8] , heme synthesis [9], free radical generation [10-12] , apoptosis [13]).

Thus, in the last years, genes responding to AZT have been identified in various cell lines [12-16]. These AZT-modulated genes code mainly for proteins related either to cell growth and/or homeostasis and metabolism. However, recent reports have shown that AZT can also activate a variety of signaling cascades (NF-kB-dependent) involved in many other functions of crucial interest for the cell life [17]. Moreover, AZT is also implicated in the oxidative damage of DNA [18-20] , in the functional impairments and structural destruction of mitochondria [12,21] and in the induction of various transcription factors [14,22-24].

The aim of this study was to extend our knowledge to AZT-regulated cellular functions by identifying gene products responsive to AZT. For this purpose we used K562 cells untreated (control), or exposed to 20 μM AZT for 3 h. This drug concentration is higher but not far from that found in the blood of AIDS patients under AZT therapy. In addition, 20 μM AZT and 3 h exposure were selected also to enhance the changes of new proteins under AZT influence, and to obtain the major differences without affecting or damaging cells growth. After two-dimensional gel electrophoresis, the protein expression profiles of these two cell samples were previously inspected and then a differential comparison based on peptide mass fingerprinting analysis was performed. Our results showed that, with respect to control cells, AZT-treated cells exhibited PDI-A3 and stathmin up-regulation (+400% and +140%, respectively). On the other hand, SOD1 and HSP-60 were found to be expressed only in control cells, while NADPK-A was evidenced in the AZT-treated samples.

## Results and discussion

To identify the proteins whose expression is responsive to AZT, we performed two-dimensional gel electrophoresis using protein extracts from K562 cells grown for 3 h in the absence or in the presence of 20 μM AZT. Master maps of control and AZT-treated K562 cells were generated by analysis with the ImageJ software [25,26] after detection of 624 spots by a medium-sensitivity stain such as micellar Coomassie Brilliant Blue G250 [27,28]. Fingerprinting by MALDI-ToF-MS analysis enabled the identification of five different spots.

**Figure 1:**
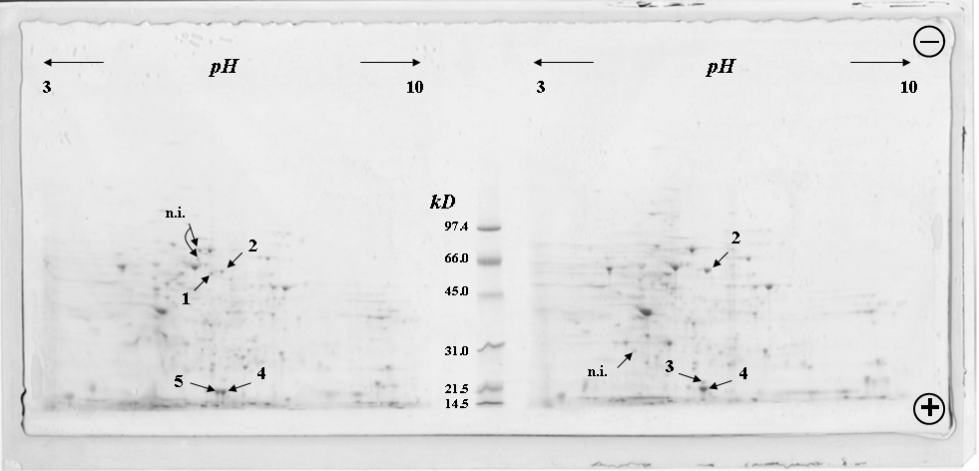
Representative Coomassie Blue G250-stained 2DE gel of K562 cells, developed in the IPG 3–10 linear interval. Left side: control K562 cells; right side: AZT-treated K562 cells. 1: HSP-60; 2: PDI-A3; 3: NDPK-A; 4: Stathmin; 5: SOD1. n.i.: not identified.

The major differences found when comparing both sets of samples, the control untreated and the AZT-treated cells, were the up-regulation of two proteins, the apparent induction of a protein and the apparent silencing expression of two proteins by AZT. Following PMF the five proteins were subsequently identified as protein disulfide isomerase A3 (PDI-A3) and stathmin (up-regulated 4 and 1.4 fold, respectively; Fig. 1, right panel); nucleoside diphosphate kinase A (NDPK-A; which was apparently present only in the treated sample; Fig. 1, right panel); cytosolic superoxide dismutase (SOD1) and 60 kDa heat shock protein (HSP-60) (both apparently present only in the control sample; Fig. 1, left panel; Table 1). The identified protein spots indicated in Fig. 1 by figure numbers (1–5) were detected in duplicate in two different sample preparations; the spots indicated in Fig. 1 by n.i. (not identified) appeared only in the representative Coomassie Blue stained 2DE gel (Fig. 1). To our knowledge, this observation is the first one that shows the direct effect of AZT on specific proteins expression.

Our results demonstrated that AZT regulated the expression of PDI-A3, an essential folding catalyst and chaperone of the ER [29-33]. This abundant protein introduces disulfides into proteins (oxidase activity) and catalyzes the rearrangement of incorrect disulfides (isomerase activity). Markovic *et al*. [34] recently confirmed a role for an oxido-reductase activity, presumably that of PDI, in the events that follow the HIV envelop binding to the membrane of cell target receptors. In fact, it has been reported that reduction of gp 120 disulfide bonds by PDI during the viral interaction with the lymphocyte surface is a strict requirement for the fusion [35,36]. However, it has been reported that PDI-A3 may regulate signaling by sequestering inactivated and activated Stat3 [37] and it is usually up-regulated following an ER stress [38,39]. In this respect, intriguingly, although AZT is known to inhibit HIV reverse transcriptase activity, it could partly favor the entry of HIV into the cell through PDI-A3. This hypothesis should be indeed appropriately supported by specific experiments which are beyond the aim of the present study.

The other modulation by AZT of protein expression identified by 2DE was stathmin (oncoprotein 18; phosphoprotein p19) which was 1.4 fold up-regulated in AZT-treated cells. This protein is a regulator of microtubule (MT) dynamics that binds tubulin heterodimers and destabilizes MTs by promoting catastrophes (i.e. transitions from growing to shrinking MTs) [40]. Interestingly, it has been found that microtubule protein contains also an enzyme, namely NDP kinase responsible for the synthesis of nucleoside triphosphates but also involved in several regulatory processes associated with cells proliferation, development, and differentiation [41]. In particular, the so-called NDPK-A, encoded by the nm23-H1 gene, is found only in the cytosol and is associated to tumour progression and metastasis. By contrast, the so-called NDPK-B, encoded by the nm23-H2 gene, is a transcription factor for c-myc and it is found both in the cytosol and in the nuclei [42]. Thus, in our experimental conditions the 1.4-fold up-regulation of stathmin and the concomitant presence of NDPK-A in AZT-treated K562 cells indicated a general effect of the drug on the microtubule system. In this context, it is worth noting that agents able to disrupt microtubules and some routes of intracellular trafficking (e.g. nocodazole, colchicine), reduce the ability of AZT to inhibit the cytotoxicity of ricin because of an alteration in membrane translocation to the cytosol [43].

The last two proteins which appeared to be expressed only in control cells, were identified as HSP-60 and SOD1, respectively. HSP-60 is localized mainly in the mitochondria [44] but it is also described to be associated with the cell membrane [45,46]. The major role of HSP-60 is its involvement in the folding of proteins during mitochondrial import; once properly folded the protein become unable to be a target for binding to HSP-60 [47]. Interestingly, it has been reported that HSP-60 interacts with gp41, a transmembrane protein anchoring the surface protein gp120 to the viral envelope of HIV; in addition gp41 mediates the fusion between the viral and the host cell membrane, a step which is essential for viral entry [48]. Since the binding of HSP-60 to gp41 enhances the infectivity of the virus by helping the virus both to anchor it to the cell surface and to escape the attack of the immune system [48], AZT could reduce the biological activity of gp41 by silencing the HSP-60 expression, thus preventing the entry of HIV into the cell. This attractive hypothesis, which is linked to gp41 through HSP-60, might counterbalance the effect of AZT on gp120 through PDI-A3, as discussed before. Furthermore, it has also been reported that HSP-60 stimulates the activity of HIV-1 retroviral integrase, an enzyme which catalyzes a critical step in the infectious cycle of this retrovirus, namely the integration of HIV-1 proviral DNA in the nuclear cell genome [49].

Concerning SOD1, the other protein which seemed to be silenced in the presence of AZT, it belongs to a family of ubiquitous enzymes, found in all aerobic cells, which are thought to provide primary defense against deleterious effects of superoxide anions (O_2 _^-^) by dismuting them to hydrogen peroxide (H_2_O_2_) and molecular oxygen (O_2_) [50]. There are two types of SOD in mammalian tissues: a Cu_2_Zn_2_SOD in the cytosol (SOD1 or SOD-c) and a Mn-SOD in the mitochondria [51]. The SOD1 was identified as spot n. 5 in our 2DE (Fig. 1). Interestingly, it has been previously found that AZT treatment of Tat mice causes almost 80–90% suppression of Mn-SOD activity [52]. Therefore, a similar effect of AZT on SOD1 can not be excluded in our experimental conditions. Since it has been reported that the extracellular SOD (EC-SOD) activity of blood plasma decreases in HIV-infected patients compared to healthy subjects, and also the SOD activity of mononuclear cells decreases with the HIV-associated disease progression [53], our results are in line with these observations and provide a further tool for a better knowledge of the AZT-induced toxicity in AIDS patients treated with this drug.

## Conclusion

In conclusion, to date, this is the first report showing over-expression of PDI-A3 and stathmin together with NDPK-A appearance, in AZT-treated K562 cells, while HSP-60 and SOD1 were detected only in control untreated cells. Of course, up/down regulation of these proteins could be not just exclusive but probably part of a larger group of proteins undetectable with our system. However, further studies are in progress extending our observations to other cell types in order to verify if the above reported alterations are a general feature of the AZT- exposed cells or they represent a peculiar behavior of a specific human chronic myeloid (K562) leukemia cell line.

## Materials and methods

### Chemicals and materials

All chemicals, but when specified were from Sigma-Aldrich (St. Louis, MO, USA). The Multiphor system as well as the linear Immobiline dry strips pH gradient 3–10 (11 cm long) were from Amersham (Milan, Italy).

### Cells and growth conditions

Human chronic myeloid (K562) leukaemia cells were obtained from the American Type Culture Collection (ATCC), maintained in exponential growth in RPMI 1640 bicarbonate medium (pH 7.2) supplemented with 10% (v/v) heat-inactivated foetal calf serum (FCS), 2 mM glutamine and 0.1 mg/mL of both penicillin and streptomycin and kept at 37°C in a humidified atmosphere of 5% CO_2 _in air. Cells, seeded at a density of 3 × 10^5 ^cells/mL, were incubated in the absence or in the presence of 20 μM AZT for 3 h in routine experiments. Other experimental conditions were also tried (from 2 to 40 μM AZT from 5 min to 48 h), but the ones reported here were those that gave both the best reproducibility of the data and the major differences between untreated (control) and AZT-treated cell samples. Therefore, a dose response or a time response curve were not included since not necessary. Cells in the exponential growth phase were harvested for the experiments. However, cell growth did not show any appreciable change at all the experimental conditions we used. Cell counting and viability were determined at various times by trypan blue exclusion method.

### Cells treatment for proteomic analysis

K562 cells, incubated alone or with 20 μM AZT for 3 h at 37°C, were washed twice with PBS (20 mM K-phosphate buffer, pH 7.2, containing 150 mM NaCl). The pelleted cells (1 × 10^6 ^cells) were then treated with 10 μL of lysis buffer (8 M urea, 2 % CHAPS, 0.3 % DTT, 2 % IPG buffer pH 3–10 (Amersham, Milan, Italy), 5 μL bromophenol blue) at 4°C for 20 min. Cell extracts were sonicated 4 times for 10 s each at the maximum power in an ice-bath and then centrifuged for 15 min at 15,000 g (4°C). The clear supernatants containing the proteins solutions were collected and stored at -80°C for no more than a week prior to the subsequent analyses. The protein concentration was determined according to Bradford [54].

### Two-dimensional gel electrophoresis (2DE)

Approximately 800 μg of each sample protein extract were treated with 2-D Clean-Up Kit (Amersham, Milan, Italy) to eliminate high levels of salt and other interfering compounds. The pellets (200 μg) were resuspended in 150 μl rehydration solution (8 M urea, 0.5 % v/v Triton X-100, 20 mM DTT, 2 % v/v IPG buffer pH 3–10) for the first dimension IEF. Eleven cm long, pH 3–10 immobilized linear pH gradient strips were rehydrated with the sample and then focused according to the method of Bjellqvist *et al*. [55]. Briefly, IPG strips were equilibrated for 10 min with IPG equilibration buffer (0.5 M Tris-HCl pH 6.8, 6 M urea, 30% glycerol v/v, 1% SDS w/v, 0.6% DTT w/v). After 10 min the procedure was repeated with another IPG equilibration buffer containing 0.3% w/v IAA instead of DTT and 5 μl bromophenol blue solution. For the second dimension, a vertical slab gel of 12.5% acrylamide was used and SDS-PAGE was performed at 20 mA per gel for 60 min at a room temperature. Gels were stained overnight with colloidal Coomassie blue (0.1% w/v Coomassie Brilliant Blue G250, 34% v/v methanol, 3% v/v phosphoric acid, and 17% w/v ammonium sulphate), while destaining was performed with a solution of 5% v/v acetic acid until a clear background was achieved [56]. Five replicas for each conditions (control and AZT-treated) were made. In addition, the same experiments were repeated twice and the spots which were constantly reproduced, as well as those which showed a differential intensity greater than 25%, were analyzed.

### Protein pattern analysis

The 2DE gels were scanned by a standard PC work station and analyzed with the ImageJ software [25,26] (Fig. 1). A match set was created from the protein patterns of the two independent cellular extracts (control K562 cells, AZT-treated K562 cells). Spot amounts of the gels were normalized to remove non-expression-related variations in spot intensity. The results were evaluated in terms of spot pixels. Statistical analysis allowed the study of proteins that were significantly increased (or decreased).

### Protein identification by mass spectrometry

Selected spots were manually excised from gels and a large number of samples were simultaneously digested with trypsin using the In-gel Digest96 Kit™ (Millipore, Bedford, MA, USA) according to the manufacturer's instructions. A minimal aliquot of the obtained tryptic peptide mixture was mixed with an equal volume of a solution of α-cyano-4-hydroxy-*trans*-cinnamic acid matrix, saturated in 50% v/v acetonitrile containing 0.1% v/v TFA, and spotted onto a MALDI target plate. Matrix-assisted laser desorption/ionization-time of flight-mass spectrometry (MALDI-ToF-MS) analyses were performed in a Voyager-DE™ STR instrument (Applied Biosystems, Framingham, MA, USA) equipped with a 337 nm nitrogen laser and operating in reflector mode. Mass data were obtained by accumulating several spectra from laser shots with an accelerating voltage of 20,000 V. All mass spectra were externally calibrated using a standard peptide mixture containing des-Arg-bradykinin (904.4681), angiotensin I (1296.6853), 1–17 (2093.0867) and 18–39 (2465.1989) adrenocorticotropic hormone fragments. Two tryptic autolytic peptides were also used for the internal calibration (*m/z *842.5100 and 2807.3145) (Table 1). Validation of mass spectrometric identifications was attempted by matching in the Swiss-2DPage database .

**Table 1:** Proteins differentially expressed in AZT-treated K562 cells.

*Spot Identification (Figure 1)*	*Protein*	*Gene name*	*SWISS-PROT accession number*	*MW (expt/pred)*	*pI (expt/pred)*	*Trend in AZT-treated cells*	*Observations and reported functions *^a^
1	HSP-60	HSPD1	P10809	61187/62000	5.70/5.75	Absent	Chaperone that accelerates the maturation of pro-caspase by upstream activator proteases during apoptosis
2	PDI-A3	GRP58	P30101	57146/63000	5.98/5.80	Increased 4.0 -fold	Chaperone in the endoplasmic reticulum lumen, may regulate signalling by sequestering inactive and activated Stat3
3	NDPK-A	NME1	P15531	17309/21000	5.83/5.90	Present in AZT-treated cells; Absent in control cells	Found in reduced amount in tumor cells of high metastasic potential. May have distinct if not opposite roles in different tumors
4	Stathmin (onco-protein 18; phospho-protein p19)	STMN1	P16949	17161/19000	5.77/5.90	Increased 1.4-fold	Microtubule-destabilizing proteins up-regulated in neoplastic cells; down-regulation in malignant cells interferes with their progression through cell cycle and abrogates their transformed phenotype
5	Cu,Zn-SOD	SOD1	P00441	16023/19000	5.70/5.75	Absent	Cellular protective function against oxidative stress. Defects in SOD1 are the cause of familial amyotrophic lateral sclerosis (FALS) also called amyotrophic lateral sclerosis 1 (ALS1 or ALS).

^a^: from SWISS-PROT entry

### Database searches

A monoisotopic mass list from each protein spot was obtained from MALDI-ToF data after exclusion of expected contaminant mass values (autolytic tryptic peptides and tryptic human keratin fragments), automatically achieved by the PeakErazor program . These peptide mass fingerprints (PMF) were used to search for human protein candidates in the SWISS-PROT database using the Mascot search engine at the site , with the following parameters: one missed cleavage permission, 50 ppm measurement tolerance and at least five matching peptide masses. Oxidation at methionine (variable modification) and S-carboxyamidomethylation at cysteine residues (fixed modification) were also considered. No post-translational modifications were allowed. Positive identifications were accepted with P values (the probability that the observed match is a random event) higher than 0.05 (Table 2).

**Table 2:** Matching parameters associated to the identified proteins from K562 cells line. (C): control; (T): AZT-treated cells.

*Spot Identification (Figure 1)*	*Protein*	*Sequence Coverage (%)*	*Score*	*Error (ppm)*
1(C)	HSP-60^a^	30	95	32
2(C) 2(T)	PDI-A3^a^	19 18	88 86	19 9
3(T)	NDPK-A^b^	42	60	7
4(C) 4(T)	Stathmin^c ^(oncoprotein 18; phosphoprotein p19)	35 18	62 44	20 7
5(C)	Cu,Zn-SOD^a^	50	67	19

^a^: matching with the liver master gel [57].

^b^: matching with the A549 cells 2D map [58].

^c^: matching with lymphocyte 2D map [59].

## Abbreviations

AZT: 3'-azido-3'-deoxythymidine or zidovudine; 2DE: two-dimensional gel electrophoresis; DTT: 1,4-dithio-DL-threitol; ER: endoplasmic reticulum; HIV: human immunodeficiency virus; HSP-60: 60 kDa heat shock protein; IEF: immunoelectrophoresis; IPG: immobilized pH gradient; MALDI-ToF-MS: matrix-assisted laser desorption/ionization-time of flight-mass spectrometry; NDPK-A: nucleoside diphosphate kinase A; PDI-A3: protein disulfide isomerase A3; PMF: peptide mass fingerprinting; SOD1: cytosolic superoxide dismutase; TFA: trifluoroacetic acid.

## Competing interests

The author(s) declare that they have no competing interests.

## Authors' contributions

GD'A heavily worked out the project, design and drafting of the manuscript; ARL has been involved in seeding, maintenance and treatment of K562 cells; SV carried out two-dimensional gel electrophoresis analyses; LDF participated in protein pattern analysis of digital images; AG performed proteins identification my mass spectrometry; GM participated in database searches; AO participated in the drafting of the manuscript; AB has been involved as an expert in AZT pharmacology and in critically revising the manuscript.
